# Is a single item stress measure independently associated with subsequent severe injury: a prospective cohort study of 16,385 forest industry employees

**DOI:** 10.1186/1471-2458-14-543

**Published:** 2014-06-02

**Authors:** Simo Salminen, Anne Kouvonen, Aki Koskinen, Matti Joensuu, Ari Väänänen

**Affiliations:** 1 Finnish Institute of Occupational Health, Occupational Safety and Ergonomics, Helsinki, Finland; 2 UKCRC Centre of Excellence for Public Health (NI), Queen’s University, Belfast, UK; 3 Department of Social Research, University of Helsinki, Helsinki, Finland; 4 University of Social Sciences and Humanities, Faculty in Wroclaw, Wroclaw, Poland; 5 Finnish Institute of Occupational Health, Creating solutions, Helsinki, Finland; 6 Finnish Institute of Occupational Health, Centre for Expertise for Work Organizations, Helsinki, Finland

**Keywords:** Stress, Injury, Forest industry, Finland, Cohort study, Hospitalisation

## Abstract

**Background:**

A previous review showed that high stress increases the risk of occupational injury by three- to five-fold. However, most of the prior studies have relied on short follow-ups. In this prospective cohort study we examined the effect of stress on recorded hospitalised injuries in an 8-year follow-up.

**Methods:**

A total of 16,385 employees of a Finnish forest company responded to the questionnaire. Perceived stress was measured with a validated single-item measure, and analysed in relation recorded hospitalised injuries from 1986 to 2008. We used Cox proportional hazard regression models to examine the prospective associations between work stress, injuries and confounding factors.

**Results:**

Highly stressed participants were approximately 40% more likely to be hospitalised due to injury over the follow-up period than participants with low stress. This association remained significant after adjustment for age, gender, marital status, occupational status, educational level, and physical work environment.

**Conclusions:**

High stress is associated with an increased risk of severe injury.

## Background

In the European Union, 22% of employees report that they suffer from stress, which in the working population is the second most common ailment after musculoskeletal disorders [1]. Injuries are also a significant health burden, accounting for 14% of global life years lost [2]. Higher levels of stress have been linked to an increased proneness to injury. A review of 20 studies showed that high stress was associated with a three- to five-fold risk of occupational injury [3]. The evidence is not unequivocal, however: a previous prospective study did not find a significant association between psychological distress and injuries among Finnish hospital personnel [4].

It is possible that the results of the earlier studies are influenced by various methodological shortcomings. The cross-sectional nature and relative short follow-ups are common limitations. Cross-sectional results leave open the possibility of reverse causality, and during a short follow-up the potential impact of stress may not have had time to appear. According to the stress literature, exposure to long-term environmental stressors in particular can cause detrimental prolonged neurohormonal reactions as well health behavioural changes [5] that may affect the risk of injury. However, in most studies stress has been measured only at one time point. Therefore it is not clear whether participants’ responses reflect a longstanding situation or just a brief, temporary reaction. Finally, most previous studies have been based on self-reported injuries. Medically verified, diagnosed injuries would offer a more reliable endpoint.

The present study used data from three time points from the Finnish Still Working Study to examine the prospective association between stress and recorded, hospitalised injuries. Employees who did not record injuries leading to hospitalisation during the preceding 2 years before the assessment of stress were followed up for 8 years.

## Methods

### Participants

**Figure 1:**
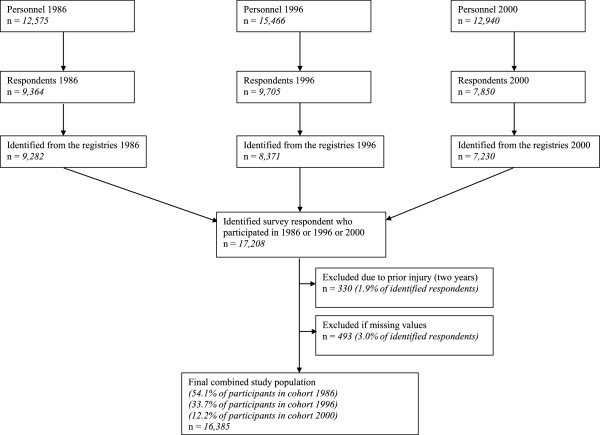
Sample selection and description of the final study population.

The data were collected as part of the ‘Still Working’ cohort study [6]. This study is based on company-wide questionnaire survey data linked to national health records. The participants consisted of the Finnish personnel of a multinational forest industry corporation.A questionnaire on psychosocial factors, health behaviours and well-being was sent to 12,575 employees of the company in Finland in 1986, to 15,466 employees in 1996 and to 12,940 employees in 2000. A total of 9282 employees responded to the questionnaire and could be identified in 1986 (response rate 76%), 8371 responded in 1996 (response rate 54%) and 7230 responded in 2000 (response rate 61%). Those who had already responded in 1986 were excluded from the 1996 cohort and those who had responded in 1986 or in 1996 were excluded from the 2000 cohort (Figure 1).

The study was approved by the Ethics Committee of the Finnish Institute of Occupational Health. Finnish laws did not require a written informed consent from the employees for this type of study. The procedure was as follows: The researchers gave each employee in the corporation an identification code, which was marked in the questionnaire. The link between this identification code and the national personal identification number given to all Finns at birth was known only to the researchers and was later used to link the questionnaire data collected in 1986 or 1996 or 2000 to data from several national Finnish health registers (e.g., injuries) until the end of 2008. Questionnaires were sent to work units, distributed to all employees and managers, and, once completed, mailed directly to the researchers. Participation was voluntary, and confidentiality was assured to all employees in the cover letter.

### Measure of stress

Stress was measured using the following question: “Stress refers to a situation where a person feels tense, restless, nervous, or anxious, or is unable to sleep at night because his/her mind is troubled all the time. Do you feel that kind of stress these days?”

1 = not at all

2 = only a little

3 = to some extent

4 = rather much

5 = very much.

The responses were categorised into three groups:

low = categories 1 and 2

intermediate = category 3

high = categories 4 and 5.

We merged the first and the second, and the fourth and the fifth category as the numbers of participants in categories 1 and 5 were very small. The present single-item stress measure has been shown to have satisfactory content, criterion and construct validity for survey research [7].

We measured repeated exposure to stress by including those participants who completed the questionnaire and responded to the stress question both in 1996 and 2000.

### Hospitalisation for injuries

Those employees who (1) were identified from the database of the National Population Register Centre; (2) were free from severe injuries (no recorded hospitalisations for injuries in the previous 2 years; this exclusion was based on the assumption that an injury experienced earlier than this would not anymore affect the new injury risk); (3) had worked for the company for at least 24 months before the survey; (4) responded to the single item stress measure; and (5) did not have missing values for any other study variables were included in the final cohort of 16,385 employees. Information on hospitalisations for injuries during the period between March 1, 1984 and December 31, 2008 was derived from the National Hospital Discharge Register. Data on hospitalisation before and after the baseline survey were linked to all respondents using each participant’s ID number.

We obtained data on all those who were hospitalised for injuries 2 years before the assessment of stress (March 1, 1984-February 28, 1986 or March 1 1994-February 28, 1996 or October 1, 1998-September 30, 2000) and 8 years after the assessment. Injuries due to vehicle and traffic injuries, falls and struck by/against were added to the measure of injury (ICD-8: E800-E859; E861-E999; ICD-9: E800-E858; E861-E999; ICD-10: V01-X59). In order to study the onset of new injuries, we excluded employees with a history of hospital admissions for injuries at baseline (n = 330). Altogether, 1332 injuries were detected during the follow-up (from March 1, 1986 to May 31, 1994 or from March 1, 1996 to May 31, 2004 or from October 1, 2000 to December 31, 2008). The mean length of follow up was 7 years and 10 months (range 0.0-8.3 years).

### Ascertainment of mortality

The dates and causes of death from 1 April 1986 to 31 December 2008 were obtained from the National Death Register kept by Statistics Finland.

### Potential confounding factors

The potential confounding factors examined were gender, age, educational level (high school vs. lower than high school), occupational status (manual vs. non-manual), marital status (married vs. not married), physical work environment: all measured at baseline. These confounders were selected because they have been associated with injury risk in earlier studies [8,9]. Data on age and gender were obtained from the National Population Register Centre, while occupational status was derived from the employer’s register. To assess the physical hazards in the work environment, we measured traditional exposures at work, such as vibration, noise, dirtiness, abnormal temperature, danger of accidents, using an 11-item check list. The format of the question was “Are the following elements present in your work environment?” (1 = I am not disturbed by it or not all; 2 = Somewhat disturbing, 3 = Very disturbing or hazardous to my health). The presence of one or more hazards indicated physically hazardous work [6].

### Statistical analysis

We analysed the prospective associations between confounding factors, stress and injuries using Cox proportional hazard regression models. Hazard ratios (HR) and 95% confidence intervals (95% CI) for categorical independent variables provided risk estimates.

The proportional hazards assumption was tested according to the method of Lin and Wei, in which the observed score process is compared with the simulated score process for each covariate. The p-value was obtained by performing a Kolmogorov-type supreme test. Because all p-values were >0.05, we can assume that the hazard was stable throughout the follow-up.

The analyses were adjusted stepwise for confounders. In model 1 age, gender, and marital status were adjusted for. In model 2 occupational status and educational level were added, whereas in the final model (model 3) physical work environment was additionally included. We tested possible interaction effects between stress and socio-demographic factors by including interaction terms in the model. Two-tailed p-values below 0.05 were considered to indicate statistical significance. We performed the analyses using SAS statistical programme package 9.1. (SAS Institute, Cary, NC).

## Results

The final analytical sample included 16,385 employees (12,561 men and 3824 women). The mean age of participants was 40.9 (SD = 9.3) years at the beginning of the study. The participants were split into two broad occupational categories: white-collar (managers, office personnel, foremen and technical staff) and blue-collar (industrial workers, maintenance staff) workers. The final study population included a higher proportion of women (23% versus 21%, p < 0.001), white-collar workers (34% versus 27%, p < 0.001), employees aged < 50 years (66% versus 52%, p < 0.0001), and those who were married (66% versus 61%, p < 0.0001) than the missing or otherwise excluded population.

Table 1 shows the characteristics of the participants at baseline, the means of stress by covariates and the Cox proportional hazard ratios for stress by the same covariates. Workers over 50 years of age were slightly more stressed than younger workers. Men suffered from stress more often than women, the participants who were married reported more stress than their non-married counterparts, and white-collar employees were significantly more stressed than blue-collar workers. In addition, higher educational level and poor physical work environment were associated with a higher level of stress.

**Table 1:** The means of stress, and age- and gender-adjusted new injury events by baseline covariates

***Background characteristics***	***N***	***No of injury events***	***Mean of stress (95% CI)***	***p-value***	***Hazard ratio (95% CI)***	***p-value***
Age				< 0.001		0.63
< 50	10750	873	2.13 (2.11-2.15)		1.00	
50+	5635	459	2.24 (2.21-3.26)		1.03 (0.92 to 1.15)	
Gender				0.047		< 0.001
Women	3824	208	2.14 (2.11-2.17)		1.00	
Men	12561	1124	2.18 (2.16-2.19)		1.68 (1.45 to 1.95)	
Marital status				< 0.001		< 0.001
Married	10892	826	2.19 (2.17-2.21)		1.00	
Not married	5493	506	2.12 (2.10-2.15)		1.26 (1.12 to 1.40)	
Occupational status				< 0.001		< 0.001
White-collar	5500	276	2.34 (2.32-2.37)		1.00	
Blue-collar	10885	1056	2.08 (2.05-2.09)		1.88 (1.65 to 2.15)	
Educational level				< 0.001		< 0.001
High school or higher	2478	123	2.28 (2.25-2.32)		1.00	
Less than high school	13907	1209	2.14 (2.13-2.16)		1.69 (1.40 to 2.04)	
Physical work environment				< 0.001		0.020
Good	9119	650	2.11 (2.09-2.13)		1.00	
Poor	7266	682	2.23 (2.21-2.25)		1.29 (1.16 to 1.44)	

Table 1 further shows that compared to women, men had a 68% higher risk of injury. Blue-collar workers and those with a lower educational level had an 88% and 69% higher risk of injury than white-collar employees and high school graduates, respectively. In addition, poor physical work environment was associated with an elevated injury risk.

Table 2 presents the results from the Cox proportional models of the association between stress and injury events. In the age, gender, and marital status adjusted model (model 1), high stress was associated with an increased risk of injury (HR 1.30, 95% CI 1.08-1.55). This association was slightly strengthened and remained significant after further adjustment for occupational status and educational level (HR 1.43, 95% 1.19-1.71) (Model 2), and additionally for physical work environment (HR 1.42, 95% 1.18-1.70) (Model 3).

**Table 2:** Association between stress and hospitalised injuries in an 8-year follow-up

		**Model 1**^**a**^	**Model 2**^**b**^	**Model 3**^**c**^
**Stress level**	**n ( cases)**	**HR (95% CI)**	**HR (95% CI)**	**HR (95% CI)**
Low	10683 (837)	1.00	1.00	1.00
Intermediate	4310 (354)	1.04 (0.92-1.18)	1.10 (0.97-1.24)	1.09 (0.96-1.24)
High	1392 (141)	1.30 (1.08-1.55)	1.43 (1.19-1.71)	1.42 (1.18-1.70)

^a^Adjusted for age, gender, and marital status.

^b^Adjusted for age, gender, marital status, occupational status and education.

^c^Adjusted for age, gender, marital status, occupational status, education, and physical work environment.

As Table 3 demonstrates, the interaction term gender × stress was not statistically significant (p = 0.96). Among women, the smaller number of participants probably widened the confidence intervals and the results became non-significant. In the analysis stratified by occupational status, high stress increased the risk of injury statistically significantly only among blue-collar employees. However, the interaction term occupational status × stress was not statistically significant (p = 0.79).

**Table 3:** Association between stress and hospitalised injuries in an 8-year follow-up, analyses stratified by gender and occupational status

***Gender***	**Men**		**Women**		
	**n (cases)/%**	**HR (95% CI)**^**a**^	**n (cases)/%**	**HR (95% CI)**^**a**^	**P for interaction**
Stress level					0.96
Low	8150 (706)/8.7%	1.00	2533 (131)/5.2%	1.00	
Intermediate	3343 (299)/8.9%	1.10 (0.96-1.26)	967 (55)/5.7%	1.11 (0.80-1.52)	
High	1068 (119)/11.1%	1.43 (1.18-1.75)	324 (22)/6.8%	1.34 (0.85-2.12)	
**Occupational status**	**Blue-Collar**		**White-Collar**		
	**n (cases)/%**	**HR (95% CI)**^**b**^	**n (cases)/%**	**HR (95% CI)**^**b**^	**P for interaction**
Stress level					0.79
Low	7486 (686)/9.2%	1.00	3197 (151)/4.7%	1.00	
Intermediate	2640 (271)/10.3%	1.12 (0.97-1.29)	1670 (83)/5.0%	1.02 (0.78-1.34)	
High	759 (99)/13.0%	1.44 (1.17-1.78)	633 (42)/6.6%	1.36 (0.96-1.93)	

^a^Adjusted for age, marital status, occupational status, education and physical work environment.

^b^Adjusted for age, gender, marital status, education and physical work environment.

Table 4 shows that the employees who experienced high stress at both measurement points with a four year interval had more than a 1.7 fold risk of severe injury during the follow-up (HR 1.74; 95% CI 1.01-2.99, in Model 2). This result attenuated slightly and became non-significant when physical work environment was included into the model (HR 1.65; 95% CI 0.96-2.84).

**Table 4:** Association between repeated exposure to stress and hospitalised injuries in an 8-year follow-up

		**Model 1**^**a**^	**Model 2**^**b**^	**Model 3**^**c**^
	**n (cases)**	**HR (95% CI)**	**HR (95% CI)**	**HR (95% CI)**
*Stress level*				
Both measurements “low” or “intermediate”	3678 (278)	1.00	1.00	1.00
Both measurements “high”	110 (14)	1.62 (0.94-2.78)	1.74 (1.01-2.99)	1.65 (0.96-2.84)

^a^Adjusted for age, gender, and marital status.

^b^Adjusted for age, gender, marital status, occupational status and education.

^c^Adjusted for age, gender, marital status, occupational status, education and physical work environment.

## Discussion

The aim of this prospective study was to examine the relationship between stress and injuries leading to hospitalisation among Finnish forest industry employees. The results showed that stress was independently associated with injuries during the 8-year follow-up period and showed a significant 30-40% increase in injury risk for all three models in those with high stress compared to those with low stress. The results are in accordance with a number of earlier studies [3]. Thus we can conclude that high stress is a risk factor hospitalised injuries.

Several possible mechanisms may explain the connection between stress and injuries. Stress has been associated with time pressure, which is a well-known risk factor in the safety literature [10]. Another intervening mechanism could be tiredness: continued stress increases tiredness, which in turn can lead to injuries [11]. Stress may also lead to carelessness, which is one of the main reasons for occupational injuries [12]. Furthermore, stress may lead to anxiety and depression, which have also been connected to a higher risk of injuries [13]. Hence, stress may prevent individual from responding appropriately to challenging or complicated tasks or situations, and stressed people may be cynical and skip phases or procedures in their actions because they do not find them worthwhile in order to invest time and energy in them.

In the present study, the association between stress and injuries was slightly stronger among blue-collar workers. The risks of occupational injury and fatality are the highest at the shop-floor level [14]. However, it is also possible that blue-collar workers’ lifestyles contain greater risks at home and during their leisure time [15]. A previous study found that industrial workers were two times more likely to experience accidents outside the workplace than at work [16].

On the basis of stress theories and meta-analyses [17] we expected that long-lasting stress will have an adverse impact of employees’ well-being and increase the risk of injury. Some of our models suggested an elevated risk of injury among those who had reported stress at two measurement points but generally speaking there was no clear significant association between repeated exposure to stress and risk of serious injury. Although the hazard ratios were rather large the wide confidence intervals and lack of statistical significance mean that these results are likely subject to type 2 error. However, it is possible that the fact that there were only a small number of injury cases (n = 14) among those who reported high stress at both measurement points has increased the confidence intervals and produced non-significant results. Additional studies are therefore needed to examine to role of chronic stress in elevating the risk of severe injuries in different populations.

Using data from the “Still Working” cohort, Ahola and her co-workers [18] showed that occupational burnout increased the risk of injury. It is not surprising that our results are in line with that study, because burnout is a chronic work-related stress syndrome. However, we used a larger dataset and had a longer follow-up period, and we measured stress in general rather than just work-related stress. Taken together, it seems that both severe work-related stress and general non-specific stress can lead to an increased risk of severe injuries that require hospital treatment.

### Strengths and limitations

Previous studies reviewed suffered from common method bias because both stress and safety outcomes were self-reported. The major strength of this study is its prospective design and a long register-based follow-up period, up to 22 years. The outcome used in the study, injury diagnosis derived from a hospital discharge register, covers all public hospital admissions in Finland, and gives an objective clinical endpoint with concrete consequences. The data on severe injuries were complete and the use of independent national register data for exclusion, adjustment, and assessment of the outcome helped us to avoid common method bias. However, our measure may be confounded by factors that influence whether or not the person seeks treatment for the injury. Our findings may provide conservative estimates, because some clinical injuries may go untreated and their effects tend to become diluted during a long follow-up period. In addition, in the final sample, male, younger, and non-manual workers were somewhat overrepresented compared to those excluded.

## Conclusions

Our single item stress measure was related to injuries to a similar extent as previous stress measures [3,18]. This is an important finding because a single item measure is easier to incorporate in occupational or other health examination questionnaires than a longer scale. The fact that reporting high level of stress was associated with subsequent injuries which required hospital treatment is important, because it helps to focus the interventions to those who could benefit the most. Around 10% of the study population experienced high stress. Prevention of injuries is possible [19]. Secondary prevention measures targeting those employees with more severe stress symptoms could be more efficient than general stress reduction programmes targeting all employees.

## Competing interests

The authors declare that they have no competing interests.

## Authors’ contributions

SS and AV together designed the study. SS directed the implementation of the study, drafted the first version of the manuscript and was the principal author of the paper. AV supervised the study, was involved in acquisition of data, contributed to design of the data analysis, interpretation of the results and manuscript writing. AKos was involved in acquisition of data, carried out the data analyses and contributed to interpreting the results and writing the paper. AKou and MJ contributed to conception and design of the study, interpretation of the results and manuscript writing. All authors read and approved the final manuscript.

## Pre-publication history

The pre-publication history for this paper can be accessed here:

http://www.biomedcentral.com/1471-2458/14/543/prepub
